# Colorimetric detection of hydrogen peroxide by dioxido-vanadium(V) complex containing hydrazone ligand: synthesis and crystal structure

**DOI:** 10.1098/rsos.171471

**Published:** 2018-03-14

**Authors:** Sunshine D. Kurbah, Ibanphylla Syiemlieh, Ram A. Lal

**Affiliations:** Centre for Advanced Studies, Department of Chemistry, North-Eastern Hill University, Shillong 793022, India

**Keywords:** colorimetric, vanadium(V) complex, crystal structure, H_2_O_2_

## Abstract

Dioxido-vanadium(V) complex has been synthesized in good yield, the complex was characterized by IR, UV–visible and ^1^H NMR spectroscopy. Single crystal X-ray crystallography techniques were used to assign the structure of the complex. Complex crystallized with monoclinic *P*2_1_*/c* space group with cell parameters *a* (Å) = 39.516(5), *b* (Å) = 6.2571(11), *c* (Å) = 17.424(2), *α* (°) = 90, *β* (°) = 102.668(12) and *γ* (°) = 90. The hydrazone ligand is coordinate to metal ion in tridentate fashion through –ONO– donor atoms forming a distorted square pyramidal geometry around the metal ion.

## 1. Introduction

Vanadium is a trace element present which exists in many oxidation states such as V(III), V(IV) and V(V). Among these three, vanadium(V) state has received considerable attention [1–3]. Depending upon the coordination mode, relative stability and basicity of the ligands vanadium switches its oxidation states between +IV and V [4,5]. The ability of vanadium to possess different interconvertible oxidation states, Lewis acidic nature and high affinity for oxygen has attracted attention of many researchers to explore its catalytic application [6,7]. Vanadium compounds play a very important role in biochemical as well as in catalytic properties. In biological systems, vanadium is present in metalloenzyme haloperoxidases that catalyse the halogenation of organic species in the presence of hydrogen peroxide [8–12]. Moreover, the metal also possesses the ability to stimulate glucose uptake, enhance insulin sensitivity, glycogen synthesis and the property to lower plasma glucose levels.

Several functional model system of vanadium haloperoxidases (VHPOs) compounds containing VO(IV)/VO(V) and/or VO_2_(V) units derived from O, N, O/S donor ligands have been successfully prepared and their structures have been established with the help of single crystal X-ray crystallography [13,14].

In this paper, we report the synthesis of a new vanadium complex containing N, O donor hydrazone ligand. The structure of complex was assigned with the help of single crystal X-ray crystallography. We also report the colorimetric sensing for hydrogen peroxide using our newly synthesized complex. Hydrogen peroxide plays a very important role as reactant both in biological systems and atmospheric chemistry [15]. Furthermore, hydrogen peroxide is of great importance in pharmaceuticals and many foods in many industries. Hence, it is important to develop an effective and convenient method for the detection of hydrogen peroxide.

## 2. Result and discussions

### 2.1. Synthesis and characterization of complex

**Figure 1.**
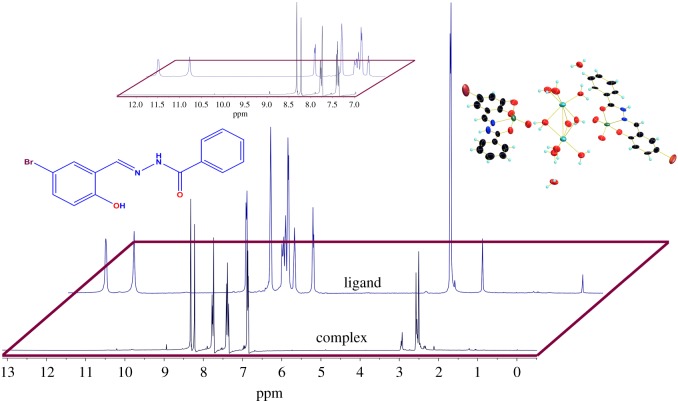
^1^H NMR of ligand and complex.

The complex was synthesized by reaction of vanadium pentoxide, sodium carbonate and the ligand in 1 : 1 : 1 molar ratio in methanol. The complex was characterized using various spectroscopic studies. The structure of the complex was assigned using single crystal X-ray crystallography. Four new prominent bands at 941, 891, 1617 and 1606 nm^−1^ were observed in the IR spectra of the complex. The bands at 941 and 891 nm^−1^ arise due to the presence of V=O and 1617 cm^−1^ is due to C=N–N=C group, whereas the band at 1606 nm^−1^ is due to coordinated azomethine –C=N functional groups [16,17]. ^1^H NMR spectrum of the complex confirmed the de-protonation of the ligand on complexation. The peak at 8.22–8.32 ppm is assigned to azomethine proton and the peaks in the 6.85–7.78 ppm range are assigned to aromatic protons (figure 1). Two absorption bands were observed in the electronic spectrum of complex. The band at 330 nm is due to intra-ligand transition, whereas the band at 420 nm is assigned to ligand to metal charge transfer (LMCT) transition that arises due to transfer of electron from p-orbital of phenolate-oxygen to an empty d-orbital of vanadium(V) centre [18].

### 2.2. Crystal structure of complex

**Figure 2.**
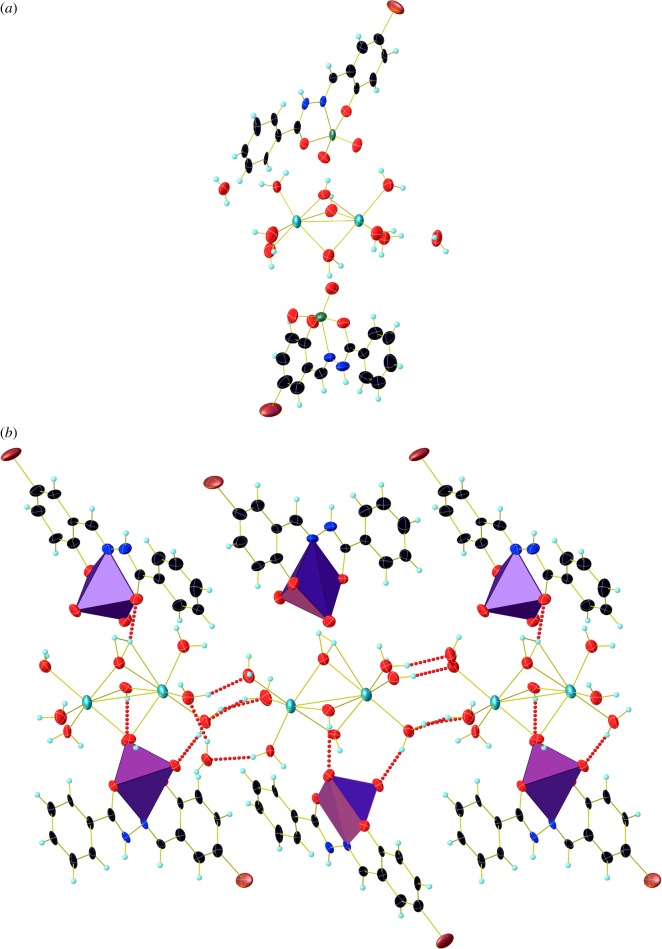
(*a*) Crystal structure of complex with 50% probability. (*b*) Molecular structure of complex showing hydrogen bonding arrangement.

The crystal data and refinement parameters of the complex are given in table 1. Crystal structure of the complex is shown in figure 2. The complex is crystallized in monoclinic *P*2_1_/*c* space group. The ligand coordinates to vanadium ion in tridentate fashion, together with two terminal oxygen group forming a square pyramidal geometry. The metal oxygen bond distances are 1.896(7) Å (V1–O1), 1.979(6) Å (V1–O2), 1.678(7) Å (V1–O3), 1.605(7) Å (V1–O4), 1.907(9) Å (V2–O5), 1.952(8) Å (V2–O6), 1.626(8) Å (V2–O7) and 1.611(9) Å (V2–O8), whereas metal nitrogen bond are 2.109(8) Å (V1–N2) and 2.158(9) Å (V2–N4). The geometrical bond angles around vanadium atom are (O3–V1–O2) 93.1(3)°, (O3–V1–O1) 97.0(3)°, (O4–V1–O3) 108.3(4)°, (O4–V1–O2) 102.3(3)°, (O4–V1–O1) 103.5(3)°, (O4–V1–N2) 102.3(3)°, (O2–V1–N2) 72.7(3)°, (O1–V1–N2) 82.9(3)°, (O5–V2–N4) 83.5(4)°, (O6–V2–N4) 75.1(4)°, (O8–V2–O5) 96.5(4)°, (O8–V2–O6) 89.8(4)°, (O8–V2–O7) 109.4(5)°, (O7–V2–O5) 102.5(5)°, (O7–V2–O6) 102.9(5)° and (O7–V2–N4) 104.0(4)°. The trans angles in the complex are (O3–V1–N2) 148.4(3)°, (O1–V1–O2) 147.6(3)°, (O8–V2–N4) 145.7(4)° and (O5–V2–O6) 150.1(4)°. The complex crystallized along with two sodium ions, in which each sodium ion bonded to six water molecules forming an octahedral geometry around the metal centre.

**Table 1.** Crystal data and structure refinement for complex.

empirical formula	C_27_H_40_Br_2_N_9_NaO_16_V_2_
formula weight	1031.37
temperature (K)	294.2(4)
crystal system	monoclinic
space group	*P*2_1_/*c*
*a* (Å)	39.516(5)
*b* (Å)	6.2571(11)
*c* (Å)	17.424(2)
*α* (°)	90
*β* (°)	102.668(12)
*γ* (°)	90
volume (Å^3^)	4203.4(10)
*Z*	4
*ρ*_calc_ (g cm^−3^)	1.630
reflections collected	26909
data/restraints/parameters	9936/0/542
goodness-of-fit on *F*^2^	1.077
final *R* indexes (*I *≥ 2*σ*(*I*))	*R*_1_ = 0.1354, w*R*_2 _= 0.2697
final *R* indexes (all data)	*R*_1_ = 0.2240, w*R*_2_ = 0.3181

### 2.3. Packing diagram of complex

**Figure 3.**
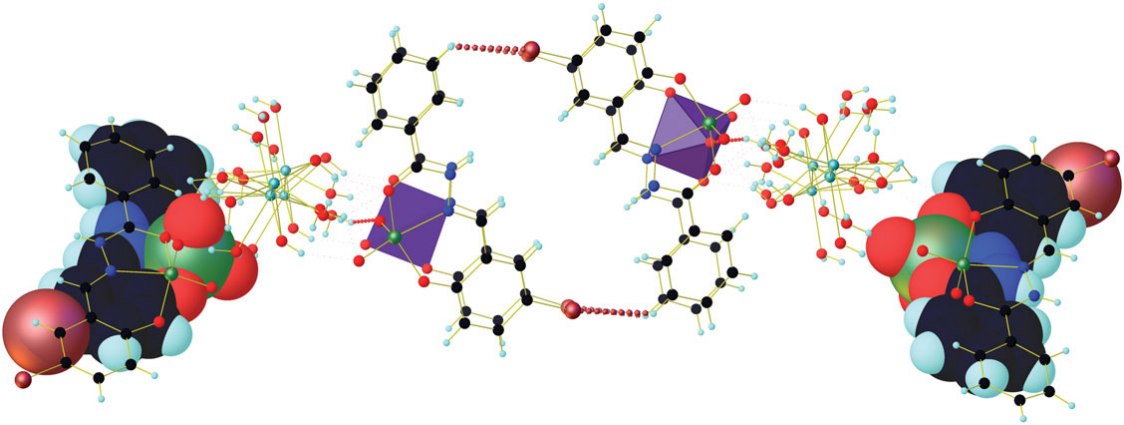
Overlay structure of complex showing inter-halogen bonding.

Molecular packing diagram of the complex consists of four molecules in a unit cell. The O–H⋯O bond lengths are O9–H9A⋯O4 (2.687(11) Å), O11–H11B⋯O19 (2.791(11) Å), O13–H13A⋯O3 (2.831(11) Å), O17–H17B⋯O18 (2.762(10) Å), whereas the bond angles are 153°, 170°, 156(7)° and 145° (figure 3). The inter-halogen bonding interactions in the complex are 3.117 Å (Br1–H11) and 3.117 Å (Br1^1^–H11^1^) (figure 3), whereas the distance between the plane of phenyl rings involved in inter-halogen bonding is 7.932 Å.

### 2.4. Colorimetric detection of H_2_O_2_

**Figure 4.**
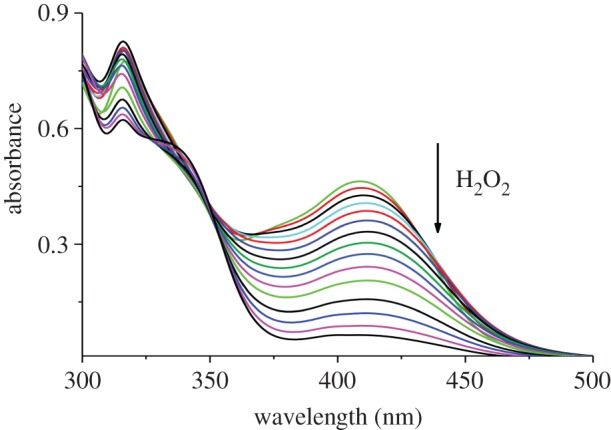
UV–visible absorption spectra of complex (50 µM) upon addition of 30% H_2_O_2_ (1 × 10^−6^ M).

**Figure 5.**
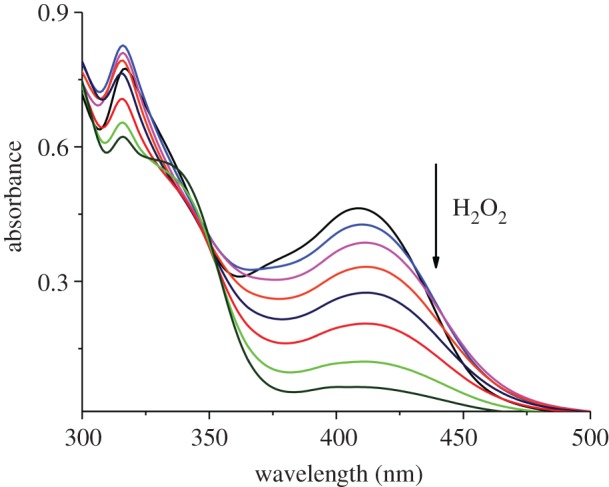
UV–visible absorption spectra of complex (15 µM) upon addition of 30% H_2_O_2_ (1 × 10^−6^ M).

**Figure 6.**
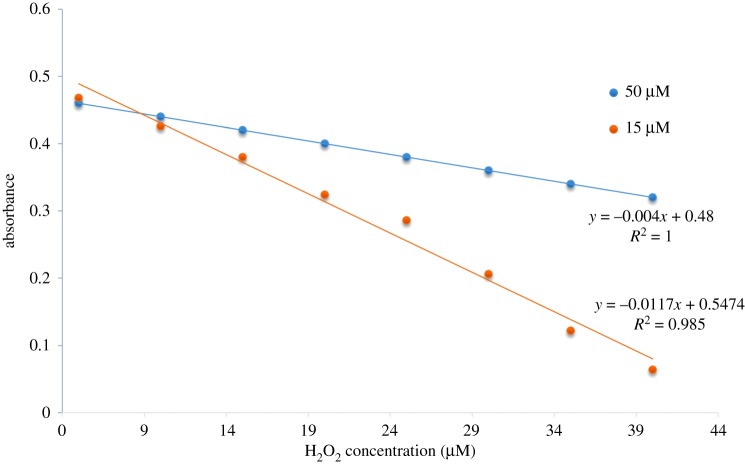
Plot of absorbance versus H_2_O_2_ at two different concentrations 50 µM and 15 µM.

In order to prove the selectivity of complex towards hydrogen peroxide, the colorimetric detection experiments were carried out in dimethylformamide solution using UV–vis spectroscopy. Upon addition of hydrogen peroxide the absorption spectra of the complex showed a dramatic change and the colour of the solution changed from yellow to dark red which could be easily detected by the naked eye. Figures 4 and 5 highlight the changes in absorption spectra upon gradual addition of hydrogen peroxide at two different concentrations (50 µM and 15 µM). Upon addition of hydrogen peroxide, the absorption band at 420 nm decreased gradually which corresponds to the reaction of complex with hydrogen peroxide. Interestingly, after addition of H_2_O_2_ nearly 2.4 equivalent, new bands at 340 nm were observed, these bands may be assigned to the formation of peroxovanadium-hydrazone species by addition of H_2_O_2_ to the solution complex, at this point of time the yellow colour of complex turned dark red [19,20]. According to the above results obtained, the complex can be used for colorimetric detection of hydrogen peroxide. The plot of absorbance versus H_2_O_2_ concentration is shown in figure 6.

Using the above titration data, the detection limit (DL) of complex as a colorimetric sensor for detection of hydrogen peroxide can be calculated using the following equation:

$$\text{DL}=\frac{KS_{d}}{m},$$

where *S*_d_ is standard deviation of blank solution, *m* is the slope of the absorbance versus hydrogen peroxide concentration and *K* is confident level. Hence, the colorimetric DL of complex for hydrogen peroxide was calculated and found to be 5.7 × 10^−6^ mol l^−1^ which could be detected the micromolar under the optimal conditions. The values obtained in the above results are comparable to those reported in the literature [21,22].

## 3. Conclusion

In conclusion, we have synthesized dioxido-vanadium(V) complex by reacting the ligand with vanadium pentoxide and sodium carbonate in 1 : 1 : 1 molar ratio in methanol solution. The complex structure was established using single crystal X-ray crystallography. The complex adopted a square pyramidal geometry with penta-coordinated structure. The colorimetric detection experiment for hydrogen peroxide was performed. DL was found to be 5.7 × 10^−6^ mol l^−1^ which could detect the micromolar quantity of H_2_O_2_. Therefore, the complex can be used for colorimetric detection of hydrogen peroxide.

## 4. Experimental section

Solvents were reagent grade and used as received. Other chemicals were E-Merck, Himedia and equivalent grades. The ligand was synthesized according to the previous reports [23,24].

### 4.1. Physical measurement

Infrared spectra in the range 4000–450 cm^−1^ were recorded as KBr discs by using a BX-III/FTIR Perkin-Elmer Spectrophotometer. The ^1^H NMR spectra were recorded on Bruker Avance II 400 and 100 MHz in DMSO-*d*_6_ solution using TMS as an internal standard. Electronic spectra were recorded on a Perkin-Elmer Lambda-25 spectrophotometer.

### 4.2. X-ray crystallography diffraction

Single crystals of the complex were obtained by slow evaporation from water/DMF solution after a few days. Data for the compound were measured using Xcalibur, Eos, Gemini diffractometer equipped with a monochromated MoK radiation (*λ* = 0.71073 Å) source. The CrysAlis PRO; Agilent, 2013 software packages were used for data collection and reduction. Absorption corrections based on multiscan use were applied [25]. The structure was solved by direct methods and refined on F^2^ by a full matrix least squares [26]. SHELXT-2014 and SHELX-2014 were used for structure solutions and refinements [27]. All non-hydrogen atoms were refined anisotropically, whereas the hydrogen atom positions were place at a calculated distance and refined freely in the final refinement.

### 4.3. Procedure for synthesis of complex

A suspension of vanadium pentoxide (0.2 g, 1 mmol) in methanol solution (10 ml) was added dropwise to a methanol solution 20 ml containing the ligand (1 mmol). The reaction mixture was refluxed for 40 min at 70°C, then cooled to room temperature for 30 min, followed by addition of sodium carbonate (0.22 g, 2 mmol) and stirred well for 20 min, till the brown colour solution turned yellow. The yellow colour solution was allowed to stand at room temperature, filtered, washed three times with warm ethanol (5 ml each time), collected and dried over CaCl_2_ to obtain the desired complex. Single crystal of complex was successfully obtained by slow evaporation of DMF solution at ambient temperature. Yield: 0.57 g (75%). M.p: > 300°C; colour: yellow. IR data (cm^−1^, KBr): 3426 (versus br) *ν*(OH), 1617, 1606 (s) *ν*(–C=N), 941, 891 (s) *ν* (VO_2_)^+^. ^1^H NMR (400 MHz, DMSO-*d*_6_, Me_4_Si): *δ* (ppm): 8.22–8.32 (d, 2H, H–C=N), 6.85–7.78 (m, 8H, Ar-H), 8.3 (s, 2H, C(H)=N). Electronic spectrum [*λ*_max_, nm]: DMF solution, *λ*_max_ (nm): 323 nm, 420 nm.

## Data accessibility

The single crystal X-ray data for this article have been deposited in the Dryad Digital Repository http://dx.doi.org/10.5061/dryad.td8kt [28].
